# Bilateral Ramsay Hunt syndrome in a diabetic patient

**DOI:** 10.1186/1472-6815-4-3

**Published:** 2004-12-02

**Authors:** Rajan Syal, Isha Tyagi, Amit Goyal

**Affiliations:** 1 Neuro-otology Unit, Department of Neuro-surgery, Sanjay Gandhi Post Graduate Institute of Medical Sciences, Raibarely Road, Lucknow (UP) – 226 014 INDIA

## Abstract

**Background:**

Herpes zoster oticus accounts for about 10% cases of facial palsy, which is usually unilateral and complete and full recovery occurs in only about 20% of untreated patients. Bilateral herpes zoster oticus can sometime occur in immunocompromised patients, though incidence is very rare.

**Case presentation:**

Diabetic male, 57 year old presented to us with bilateral facial palsy due to herpes zoster oticus. Patient was having bilateral mild to moderate sensorineural hearing loss. Patient was treated with appropriate metabolic control, anti-inflammatory drugs and intravenous acyclovir. Due to uncontrolled diabetes, glucocorticoids were not used in this patient. Significant improvement in hearing status and facial nerve functions were seen in this patient.

**Conclusions:**

Herpes zoster causes severe infections in diabetic patients and can be a cause of bilateral facial palsy and bilateral Ramsay Hunt syndrome. Herpes zoster in diabetic patients should be treated with appropriate metabolic control, NSAIDS and intravenous acyclovir, which we feel should be started at the earliest. Glucocorticoids should be avoided in diabetic patients.

## Background

Varicella-zoster virus, member of Herpesviridae family has structural characteristics like a lipid envelope surrounding a nucleocapsid with icosahedral symmetry, a total diameter of 180–200 nm and centrally located double-stranded DNA about 125,000 bp in length. Varicella-zoster virus lies latent in sensory root ganglion for years in a patient who had chickenpox earlier. Some precipitating factor may reactivate it especially when immunity of patient wanes leading to Herpes zoster, a sporadic disease. Involvement of geniculate ganglion of sensory branch of facial nerve leads to Herpes zoster oticus also known as Ramsay Hunt syndrome. Involvement of facial nerve leads to otalgia, lower motor neuron homolateral facial paralysis and vesicular eruptions in auricle and external auditory canal. In severe cases of herpes zoster oticus, involvement of vestibulocochlear nerve leads to sensorineural hearing loss in 10% and vestibular symptoms in 40% patients. Herpes zoster oticus accounts for 10% cases of the facial palsy, paralysis is usually complete and full recovery occurs in only about 20% of untreated patients [1].

Herpes zoster rash is characterized by unilateral vesicular eruptions with in a single dermatome. In 16% of patients of zoster, vesicles develop beyond single dermatome [1]. Onset of disease is heralded by pain with in dermatome that may precede lesions by 48–72 hrs; total duration of disease is 7–10 days. In immunocompromised and elderly patients course of herpes zoster is more prolonged and severe. Rarely in such patients zoster may successively involve further dermatomes.

Considering, rarity of bilateral herpes zoster, a diabetic patient presenting with bilateral Ramsay Hunt syndrome is being reported here.

## Case presentation

Diabetic male, 57 year old presented to us with history of pain in left ear for the last 8 days. 48–72 hrs after the onset of otalgia, patient developed facial weakness on left side along with vesicular eruptions on left conchae and in left external auditory meatus. After another 24–48 hrs patient had similar episode on right side. On the day of reporting to us patient was having bilateral facial weakness, impaired taste sensation, dryness of eyes along with decreased hearing on both sides but there was no history of vertigo or any ear discharge. There was history of stressful life events in past 6 months before the onset of rash.

**Figure 1:**
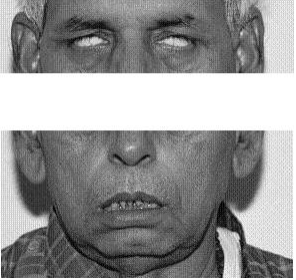
Patient at time of presentation, photograph showing bilateral lower motor neuron type of facial palsy and presence of Bell's phenomenon.

On examination, there was bilateral lower motor neuron facial palsy which was complete. Bell's phenomenon was present on both sides Fig-1. There were adherent crusts and scabs in left conchae and external auditory meatus. While vesicular eruptions were present in right external auditory meatus. Tuning fork tests were showing bilateral sensorineural hearing loss.

The patient was admitted and investigated. His postprandial blood sugar was 339 mg%, HbAIc was 7%. Pure tone audiometery was showing mild to moderate bilateral sensorineural hearing loss, stapedial reflexes were absent on both sides on tympanometery. There was impaired taste sensation from anterior two third of tongue. ELISA and Western Blot tests for HIV infection were negative. Liver function tests, tumor markers, thyroid hormones; serum ACE levels were all with in normal limits. Lumbar puncture revealed normal pressure. Glucose, protein and white blood cell count were all with in normal limits in CSF. Plain X-ray views of mastoid, internal auditory meatus and chest were normal. Computerized tomography of brain stem, cerebellopontine angle, temporal bone and skull base were normal. A smear from floor of vesicle stained with Giemsa stain showed degenerating cells with multiple nuclei. This favoured the clinical diagnosis of herpes zoster oticus. This diagnosis was confirmed by detection of IgM antibodies to Varicella-Zoster virus by ELISA test.

**Figure 2:**
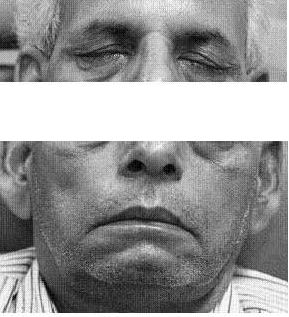
Patient after 8 wks of follow up, photograph showing complete closure of eyes.

Diabetes of this patient was controlled with insulin. Intravenous acyclovir was given in dose of 10 mg/kg every 8 hr for 7 days. Glucocorticoids were avoided in this patient due to diabetes, but NSAIDS were given. After 2 weeks of treatment and diabetes control, pure tone audiometery showed improvement in hearing by 10 db in all frequencies. 8 weeks later in the follow up, patient was able to close his eyes completely Fig-2. and facial nerve functions on both sides recovered; recovery was more on right side.

## Discussion

Severity and incidence of herpes zoster increases in elderly and in immunomodulated state like in AIDS, lymphoproliferative disorders, disseminated carcinomatosis, diabetes, during steroid therapy, during radio or chemotherapy [2]. Kubeyinje EP.(1995) found that varicella runs more aggressive course in diabetic patients as compared to otherwise healthy individuals [3]. Neu I et al (1977)in their study found basal metabolic disorders especially diabetes of particular significance inactivation and in primary and secondary manifestations of varicella zoster virus [4]. Muller C. et al (1989) concluded that metabolic derangement in diabetes leads to reversible disturbance in certain cellular immune functions which can be normalized by good metabolic control achieved by insulin treatment [5]. Postprandial blood sugar of our patient was 330 mg% at time of presentation and HbA_1c _was 7.0%, so blood sugar control was not appropriate in this patient. Patient was also under severe psychological stress since last 6 months and psychological stress is identified as a potential risk factor for zoster that might operate by suppressing cell-mediated immunity [6]. So uncontrolled diabetes and psychological stress were two risk factors present in this patient, making him prone for severe and recurrent infection of herpes zoster.

Hiroshige K et al (2002) conducted a study based on detection of varicella zoster virus DNA in tear fluid and saliva of patients with Ramsay Hunt syndrome and concluded that varicella zoster virus reactivation occurs in the unaffected side at the same level as in the affected side [7]. This explains the occurrence of bilateral Ramsay Hunt syndrome due to herpes zoster oticus in this patient. Shoji H et al (1980) also suggested that in Ramsay Hunt syndrome and its subgroups, bilateral involvement or wide spread of infection through nervous tissue can occur though its incidence is very rare[8].

In the management part, as metabolic control of diabetes improves leukocyte functions and overall immune status of patient [5] so insulin was started and dose was adjusted to achieve good metabolic control. As incidence of severe and disseminated infections of Herpes zoster is more in diabetes so intravenous acyclovir in dose of 10 mg/kg every 8 hr for 7 days was given. Recent reports suggest that treatment with i.v acyclovir decreases the incidence of permanent facial nerve palsy in Ramsay Hunt Syndrome. Results of relevant control trials have not yet been published [9]. Glucocorticoid therapy usually used in facial palsy due to herpes zoster oticus was avoided in this patient due to uncontrolled diabetes but NSAIDS and other analgesics were given in this patient.

On follow up of this patient, facial nerve recovered on both sides, recovery was more on right side. This was documented by electroneurography which showed greater amplitude of muscle compound action potentials on right side as compared to left. This might have been due to lesser time gap between the onset of acyclovir therapy and attack of zoster oticus on right side. Time gap between the onset of acyclovir therapy and attack of zoster oticus is most relevant prognostic factor in recovery of these patients and this hypothesis is supported by findings in studies of Mcgrath N. (1997) [10], Raschilas F (2002) [11] and Strupp M. et al (2004) [12]. There was also a significant improvement in hearing status of patient. This result was in accordance with other studies which keep age 64 years or younger, mild initial hearing loss, a cochlear pattern of hearing loss and absence of vertigo as factors favorable for recovery of auditory function [13].

## Conclusions

Herpes zoster can cause severe infections in diabetic patient and can cause bilateral facial palsy and bilateral Ramsay Hunt syndrome.

-Herpes zoster in diabetic patients should be treated with appropriate metabolic control, NSAIDS and intravenous acyclovir, which we feel should be started at the earliest.

-Intravenous acyclovir therapy in cases of herpes zoster oticus is effective in control of disease and prevents the incidence of permanent facial palsy but treatment should be started early in the course of disease preferably with in 72 hrs from start of disease.

-Glucocorticoids should be avoided in Herpes zoster patients having uncontrolled diabetes.

## Competing interests

The author(s) declare that they have no competing interests.

## Authors' contributions

All three authors

1) Have made substantial contributions in management of this case and in conception, design, analysis and interpretation of results of this case report

2) Have been involved in drafting the article or revising it critically for important intellectual content.

3) Have given final approval to the version to be published.

## Pre-publication history

The pre-publication history for this paper can be accessed here:


